# Cloning, Expression, and Tobacco Overexpression Analyses of a *PISTILLATA*/*GLOBOSA*-like (*OfGLO1*) Gene from *Osmanthus fragrans*

**DOI:** 10.3390/genes12111748

**Published:** 2021-10-30

**Authors:** Zhanghui Zeng, Si Chen, Mingrui Xu, Min Wang, Zhehao Chen, Lilin Wang, Jiliang Pang

**Affiliations:** College of Life and Environmental Sciences, Hangzhou Normal University, Hangzhou 310036, China; zhzeng@hznu.edu.cn (Z.Z.); chen_si0101@mingmatechs.com (S.C.); Xumingrui666@163.com (M.X.); wangminmm2021@163.com (M.W.); zhchen@hznu.edu.cn (Z.C.); wang_208@163.com (L.W.)

**Keywords:** *OfGLO1*, *Osmanthus fragrans*, B-class MADS-box, ectopic expression, flower organ development

## Abstract

*GLOBOSA* (*GLO*), a B-class MADS-box gene, is involved in floral organ determination but has rarely been studied in *Osmanthus fragrans*, which is a very popular ornamental tree species in China. Here, the full-length cDNA of a homologous *GLO1* gene (named *OfGLO1*) was cloned from a flower bud of *O. fragrans* using the RACE technique. The *OfGLO1* has a 645 bp open reading frame, encoding 214 amino acids. Similar to other PI/GLO proteins, OfGLO1 has two conserved domains, MADS MEF2-like and K-box, and a 16-amino-acid PI motif in the C terminal region. Our phylogeny analysis classified OfGLO1 as a PI-type member of the B-class MADS-box gene family. The qRT-PCR assay showed that the expression of *OfGLO1* in *O. fragrans* was continuously upregulated from the tight bud stage to the full flowering stage but barely expressed in the pistils, sepals, and non-floral organs, such as root, leaf, and stem. The genetic effect of *OfGLO1* was assayed by ectopic expression in tobacco plants. Compared with the wild-type, *OfGLO1* transformants showed reduced plant size, earlier flowering, shorter stamens, and lower seed setting rates. Furthermore, some stamens were changed into petal-like structures. These findings indicate that *OfGLO1* plays an important role in the regulation of flower development. This study improved our understanding of class B gene function in woody plants.

## 1. Introduction

Flowers of most higher-order plants consist of four whorls: sepals, petals, stamens, and carpels. The development of a floral organ is controlled by floral organ identity genes. The temporal and spatial expression patterns of these identity genes in cells determine the shape of the floral organ. Based on molecular and genetic studies in two model plants, *Arabidopsis thaliana* and *Antirrhinum majus*, Coen and Meyerowitz proposed a classical ABC model [1] to predict whether three classes of homeotic genes (encoding the A, B, and C functions) act alone or in combination to give rise to sepals, petals, stamens, and carpels. This prediction can be confirmed to some extent. However, with the identification of more flowering mutants and their corresponding genes, many phenomena could not be explained using the ABC model [2]. Subsequently, other models, such as ABCDE [3], tetramer [4], boundary attenuation [5], boundary slip [6,7], and BC bifunctional genes [8], were derived. Most of the floral organ identity genes contain an MCM1/AGAMOUS/DEFICIENS/SRF (MADS)-box, which plays a crucial role in the emergence of flower structures during plant evolution [9,10,11,12]. The class B MADS-box genes in angiosperm are essential for determining petal and stamen identity and include two evolutionary lines: *APETALA3*/*DEEICIENS* (*AP3*/*DEF*) [13] and *PISTILLATA*/*GLOBOSA* (*PI*/*GLO*) [14]. Functional mutations of these genes result in obvious floral homeotic mutants, in which the petals are transformed to sepals and the stamens are replaced by carpels [14,15,16]. The *PI/GLO* genes have been cloned from diverse angiosperm species, including *Oryza stative* [17], *Zea mays* [18], *Tulipa gesneriana* [19], *Asparagus officinalis* [20], *Phalaenopsis equetis* [21], *Lilium longiflorum* [22], *Cymbidium goeringii* [23], *Camellia sinensis* [24], *Gerbera hybrid* [25], *Canna indica* [26], etc., and they have been confirmed to play an essential role in determining petal and stamen identity.

The rapid development of biotechnology provides possibilities and developmental opportunities for people to engineer the shape, color, and aroma of the flower and the flowering time. The study of floral development genes can not only improve our knowledge of floral development but also guide practical applications to improve human quality of life. Current molecular models used to explain floral development were established based mainly on herbaceous plants such as *Arabidopsis* and *Antirrhinum*. Generally, woody plants have longer growth cycles, higher heterozygosity, and larger genetic loads than those of herbaceous plants. Therefore, these models may be limited in their explanation of floral organ development in woody species. In tea plants (*Camellia sinensis*), the expression patterns of *CsGLO1* and *CsGLO2* revealed that B-class genes were expressed not only in petals and stamens but also in sepals, which are the first round of flower organs [24]. Louati et al. [27] isolated a *PI*-like B-class gene from the Argan Tree (*Argania spinosa*) and showed that this gene was involved in petal and stamen identity. In addition, in situ hybridization analysis revealed that *BpMADS2*, a birch homolog for *PI*, was expressed in stamens and carpels of birch inflorescences [28]. Overall, the mechanisms underlying the regulation of B-class genes in flower development in woody plants are limited.

*Osmanthus fragrans* (Oleaceae) is an important economic tree species in China. Due to its ornamental, edible, and medicinal value, its flowers have long been the focus of interest in studies of *O. fragrans*, which includes the flower color [29], the floral fragrance, and the compounds [30,31,32]. However, information on flower development is still limited. Few reports focus on the B-class floral organ development genes in *O. fragrans*. In this study, we aimed to characterize the homologous *GLO1* gene in *O. fragrans*. Osmanthus floral buds were used as the test material, the full length of the *GLO1* homologous gene (*OfGLO1*) was cloned using the RACE technique, and the characteristics of the expression of *OfGLO1* were analyzed using qRT-PCR. The transgenic overexpression of *OfGLO1* was assessed in tobacco (variety ‘yellow seedling fish’) mediated by *Agrobacterium tumefaciens*, and the potential role of *OfGLO1* in floral development was analyzed and discussed.

## 2. Materials and Methods

### 2.1. Plant Materials

The sweet-scented Osmanthus (*Osmanthus fragrans*, variety ‘thunbergii’) planted on the campus of Hangzhou Normal University was taken as the experimental material. The florescence of *O. fragrans* was divided into four stages: the tight bud stage (the flowers are not yet opened and are pale yellow), the early flowering stage (the flowers open slightly, and the color turns darker), the full flowering stage (the flower opens to its maximum degree, and the color is a golden yellow), and the late flowering stage (brown spots start to appear on the petals, and the petals begin to fall off). Samples of the flowers were collected from each of the four stages. The flowers in the full flowering stage were divided into petals, stamens, pistils, and receptacles with sepals (the length of the sepal was less than 1 mm (Figure S1), too small to separate out). Finally, the samples collected were frozen immediately in liquid nitrogen and kept at −80 °C until RNA isolation.

Tobacco seeds (variety ‘yellow seedling fish’) were surface-sterilized using 3% H_2_O_2_ for 30 min and then were thoroughly rinsed in Milli-Q water before being placed on a piece of wet filter paper in a 10 cm Petri dish. The Petri dishes were wrapped and sealed tightly with parafilm and placed inside a growth chamber for ten days in a 16 h photoperiod and day/night temperatures of 28/24 °C. The seed germination rate was calculated at the end of the tenth day.

### 2.2. Extraction of Total RNA and Reverse Transcription

The total RNA was extracted using TRIzol-Reagent (Invitrogen, Carlsbad, CA, USA) according to the manufacturer’s instructions. Subsequently, one microgram of total RNA was digested with a gDNA Remover Kit and reversely transcribed into cDNA in the ReverTra Ace qPCR RT Master Mix (Toyobo, Kyoto, Japan) in a 20 µL reaction volume. The resultant cDNA was used directly as the template for the next PCR reaction.

### 2.3. OfGLO1 Gene Cloning from O. fragrans

Primers GLO1-F and GLO1-R (Table S1) were designed according to the partial CDS sequence (HQ853419) of the GLO1 gene of *O. fragrans* published on NCBI [33] for PCR amplification. The PCR reaction system consisted of cDNA at 2 μL, 2× Trans HiFi Mix at 12.5 μL, GLO1-F and GLO1-R at 1 μL, and ddH2O added at 25 μL. The reaction procedure is as follows: 94 °C pre-denaturation for 4 min; 94 °C denaturation for 30 s, 55 °C annealing for 30 s, 72 °C extension for 30 s, and amplification of 30 cycles; and 72 °C extension for 8 min. The intermediate fragment was purified using the fast gel DNA mini purification kit (Vazyme Biotech, Nanjing, China) and then was connected to the pEASY-T5 Zero vector, which was thermally transformed to *Escherichia coli* Trans1-T1 competent cells, and single colonies were selected for plasmid isolation and DNA sequencing (Sunny Biotech, Shanghai, China).

The amplification and synthesis of 3′ RACE cDNA were conducted according to the instructions of the First Choice^®^ RLM-RACE Kit (Ambion, Austin, TX, USA), with minor modifications. In brief, 1 µg of RNA, 4 µL of a dNTP Mix, 2 µL of the 3′ RACE Adapter, 1 µL of DMSO, and 8 µL of RNase-free water were mixed and incubated at 65 °C for 5 min and in an ice bath for 2 min, and then the following components were added: 2 µL of a 10× RT buffer, 1 µL of an RNase inhibitor, and 1 µL of an MMLV reverse transcriptase. After incubating at 42 °C for 1 h, the resultant reaction solution was 3′ RACE cDNA. According to the intermediate fragment sequence, two 5′ end nested primers (Table S1) were designed to be nested with the primers introduced by the RACE kit and subjected to two rounds of PCR amplification. The PCR product was recovered, ligated, and identified by DNA sequencing (Sunny Biotech, Shanghai, China).

The synthesis and amplification of 5′ RACE cDNA were carried out according to the specifications of the First Choice^®^ RLM-RACE Kit (Ambion, Austin, TX, USA), as follows: first, the total RNA was dephosphorylated using Calf Intestine Alkaline Phosphatase (CIP); second, CIP-treated RNA was treated using Tobacco Acid Pyrophosphatase (TAP); third, the CIP/TAP-treated RNA was ligated; and finally, the ligated RNA was reversely transcribed, and the resultant reaction solution was 5′ RACE cDNA. According to the intermediate fragment sequence, two 3′ end nested primers (Table S1) were designed to be nested with the primers introduced by the RACE kit and subjected to two rounds of PCR amplification. The PCR product was also confirmed by DNA sequencing.

The three-stage sequence (intermediate sequence, 3′ sequence, and 5′ sequence) obtained by splicing with SeqMan was used to predict the open reading frame (ORF) sequence. Primers with restriction sites were designed near the start codon and stop codon (Table S1). Additionally, the full-length ORF was amplified by PCR using the cDNA of *O. fragrans* as a template and confirmed by DNA sequencing.

### 2.4. Bioinformatics Analysis of Sequences

The nucleotide sequence alignment, amino acid sequence alignment, sequence splicing, ORF prediction, and domain analysis were performed on the NCBI website (http://www.ncbi.nlm.nih.gov/, accessed on 6 March 2017) using the SeqMan computer program; the isoelectric point and molecular weight of the encoded protein were predicted using Compute pI/Mw software (http://web.expasy.org/compute_pi/, accessed on 1 June 2019); the signal peptide cleavage site was predicted using SignalP online software (http://www.cbs.dtu.dk/services/SignalP-4.1/, accessed on 5 June 2019); and the multi-sequence alignment and the construction of phylogenetic tree were performed using MEGA6.6 software. In addition, gene promoter analysis was performed using the plantCARE website (http://bioinformatics.psb.ugent.be/webtools/plantcare/html/, accessed on 26 October 2021).

### 2.5. Organ-Specific Expression Analysis Using Quantitative Real-Time RT-PCR

A transcript analysis was conducted using quantitative real-time RT-PCR similar to that described by [34]. The cDNA of different floral organs at the full flowering stage and petal samples at different development stages were diluted 20-fold and amplified on a quantitative real-time PCR instrument (Bio-Rad CFX96, Hercules, CA, USA). The reaction systems were prepared with an AceQ Universal SYBR Green qPCR Master Mix kit (Vazyme Biotech, Nanjing, China) following the manufacturer’s instructions. Three biological replicates were used for transcript analysis, and three technical replicates were conducted for each cDNA sample. The constitutive expression of the cytokine gene (*qActin* for *O. fragrans*, *β-Actin* for tobacco plants) was used as the internal reference gene, and the relative expression levels were analyzed by the 2^−ΔΔCt^ method. The primer sequences for all genes determined are listed in the Supplementary Materials Table S1.

### 2.6. Vector Construction and Tobacco Transformation of OfGLO1

The vector pEASY-T5 Zero with full-length cDNA of *OfGLO1* was double-digested with the enzymes of *Kpn* I and *Xba* I and then connected to the pCAMBIA13011 vector, which contained a 35S promoter. The resultant *35S::OfGLO1* expression vector was introduced into *Agrobacterium tumefaciens* GV3101 by the heat shock method. The tobacco (variety ‘yellow seedling fish’) was transformed using a leaf disc and double screened by 30 mg·L^−1^ hygromycin B and 60 mg·L^−1^ kanamycin. Resistant seedlings were detected by PCR for positive seedlings, transplanted into the flower pot, and cultured in an artificial climate chamber with the following conditions: 24 °C for 12 h during daytime and 18 °C for 12 h at night. The culture medium consisted of a peat-to-perlite ratio of 3:1. The transgenic tobacco seedlings (T_0_ generation) were transplanted for 30 days, and the leaves of the 25 transgenic tobacco plants were analyzed using RT-PCR. The growth status and floral organ phenotype were observed and compared with the wild-type tobacco plants.

## 3. Results

### 3.1. Sequence of the OfGLO1 Gene

According to a partial cDNA sequence (GeneBank accession ID: HQ853419) of the GLO1 gene of *O. fragrans* [33], the primers were designed for PCR amplification. A 345 bp intermediate fragment was obtained using the cDNA of floral buds of the *O. fragrans* as a template (Figure S2a). Target fragments of 610 bp (Figure S2b) and 481 bp (Figure S2c) at the 3′ and 5′ ends were obtained using the RACE method, respectively. Finally, a full-length 876 bp cDNA sequence, named *OfGLO1* (GeneBank accession ID: KY575409), was obtained by sequence splicing.

**Figure 1:**
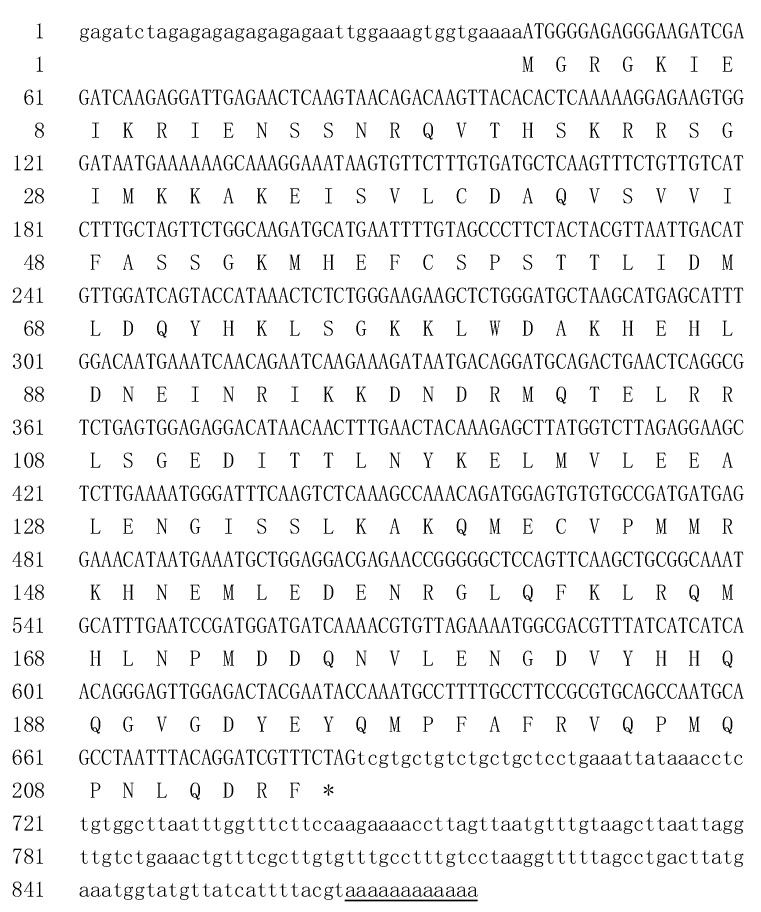
cDNA sequence of *OfGLO1* and its deduced amino acid sequence. Note: * means a stop codon. The underlined sequence is the typical tail signal poly (A). The sequence information for *OfGLO1* has been submitted to the GenBank database. The accession number is KY575409.

The sequence analysis showed that *OfGLO1* had a 645 bp opening reading frame (ORF) (Figure 1), with a translation initiation codon (ATG) and a stop codon (TAG). The third base upstream from the start codon ATG was A, and the base after ATG was G, conforming to the Kozak rule: A/GNNATGG. The 3′ end noncoding region was 191 bp, which contained the typical tail signal poly (A). The 645 bp ORF encoded 214 amino acids with a molecular weight of 2.5 kD. The isoelectric point of the predicted protein was calculated at 7.13. Further analysis revealed that OfGLO1 did not have a signal peptide splicing site at the N-terminal. Based on the recent release of *O. fragrans* genome sequence [29], the gene structure of *OfGLO1* was analyzed. The annotated *OfGLO1* gene is 2233 bp in full length, with seven exons and six introns (Figure S5). Promoter analysis of 3-kb 5′upstream fragment of *OfGLO1* identified four putative CArG boxes (consensus sequence: CC(A/T)_6_GG) located at −2775 to −2765; −2501 to −2491; −1427 to −1418; −153 to −144, relative to the translation start site (Table S2). However, all of these have a conversion of one nucleotide compared with the CArG box consensus sequence, which has also been found in the promoter of birch *PI*/*GLO* [28]. In addition, an eight bp motif (TCAAGAAA, *PI* late expression element [35]), involved in regulating *PI* expression, was identified at −650 to −643 (Table S2).

### 3.2. Sequence Alignment and Phylogenetic Analysis of GLO Homologs among Different Species

**Figure 2:**
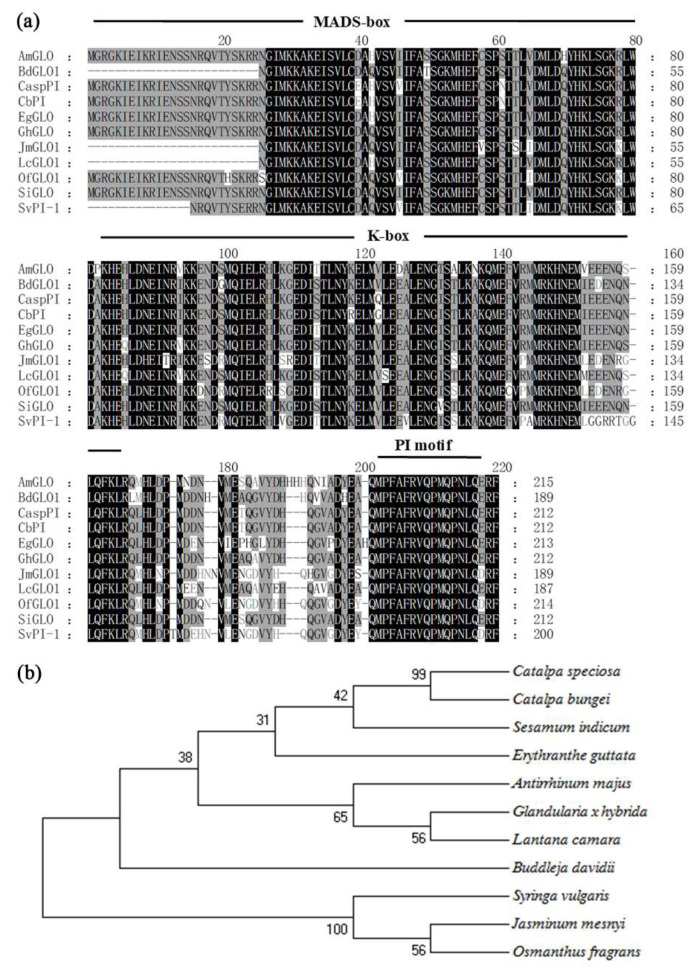
Sequence alignment and phylogenetic analysis of GLO homologs among different species. (**a**) Multiple alignment of amino acids. (**b**) Phylogenetic analysis. Completely identical amino acids are indicated with white letters against a black background; conservative amino acids are indicated with black letters against a grey background; the partly similar and non-similar amino acids are indicated by grey letters and black letters on a white background, respectively. The MADS-box, K-box, and PI motif are indicated by lines. The phylogenetic tree was constructed using the Neighbor-Joining method in MEGA6.6. The amino acid sequences were aligned as follows: AmGLO (*Antirrhinum majus*, Q03378); BdGLO1 (*Buddleja davidii*, AEM60219); CaspPI (*Catalpa speciosa*, AMB20847); CbPI (*Catalpa bungee*, AJY60427); EgGLO (*Erythranthe guttata*, XP_012855839); GhGLO (*Glandularia x hybrid*, BAE72882); JmGLO1 (*Jasminum mesnyi*, AEM60214); LcGLO1 (*Lantana camara*, AEM60216); OfGLO1 (KY575409, *Osmanthus fragrans*); SiGLO (*Sesamum indicum*, XP_011081947); and SvPI-1 (*Syringa vulgaris*, AAC42576).

The amino acid sequence alignment of OfGLO1 with other species of homologous proteins showed that the MADS MEF2-like and K-box, typical features of the MADS-box type II gene, were highly conserved. The MADS MEF2-like structure and the K-box structure in OfGLO1 are located at amino acids 2–80 and at amino acids 83–163, respectively (Figure 2a). In addition, a 16-amino-acid PI motif (MPFAFRVQPMQPNLQE) was found in the C terminal region, indicating that OfGLO1 is a PI/GLO-type member of the class B MADS-box gene. The homology search showed that OfGLO1 had the highest similarity to JmGLO1 from *Jasminum mesnyi,* with a homology of 91%, followed by 83% with BdGLO1 in *Buddleja davidii* and 81% with LcGLO1 in Lantana. Other GLO proteins from sesame, *Verbena*, monkey flower, and snapdragon had 86%, 85%, 85%, and 82% homologies, respectively, with OfGLO1.

To study the evolutionary relationship between the OfGLO1 and PI/GLO proteins of other plants, a phylogenetic tree was constructed using the Neighbor-Joining (NJ) method (Figure 2b). The results showed that *O. fragrans*, *Jasminum mesnyi*, and the lilac poly of Oleaceae clustered together, presenting the closest relationship, consistent with species phylogeny classification.

### 3.3. Organ-Specific Expression Analysis of OfGLO1 Gene

**Figure 3:**
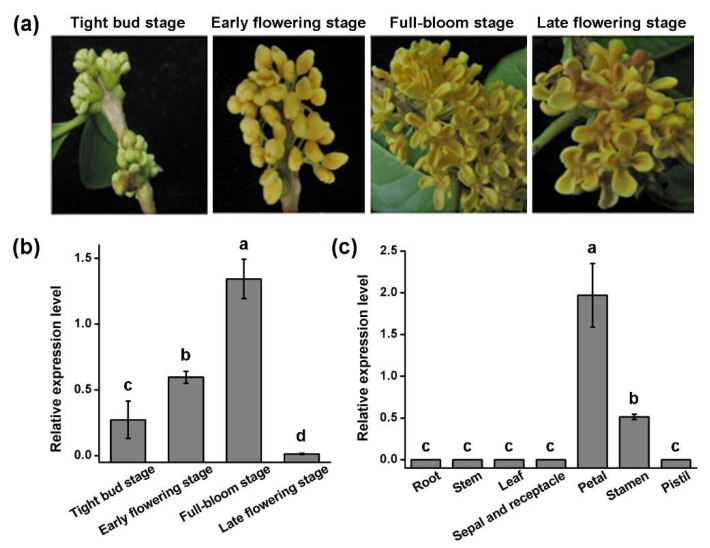
Expression profile of *OfGLO1* in different flower organs of *O. fragrans*. (**a**) The phenotypes of *O. Fragrans* flowers at four different floral stages. (**b**) The expression levels of *OfGLO1* in *O. Fragrans* flowers at four different floral stages. (**c**) The expression levels of *OfGLO1* in different tissues of *O. fragrans* at the full-bloom stage. Quantitative real-time RT-PCR was used to determine the relative expression levels, and cytokine gene (*qActin*, Table S1) was used as the internal reference gene. The different letters indicate significant differences according to ANOVA followed by Duncan’s test (*p* < 0.05). Data are means (±SD) from three biological replicates.

The expression profile of *OfGLO1* in different floral stages, including the tight bud stage, the early flowering stage, the full-bloom stage, and the late flowering stage, was determined using quantitative real-time RT-PCR (Figure 3a,b). The results showed that the expression of *OfGLO1* in *O. fragrans* was continuously upregulated from the tight bud stage to the full flowering stage but barely expressed in the late flowering stage (Figure 3b). *OfGLO1* had the highest expression at the full-bloom stage (Figure 3b). At the full-bloom stage (Figure 3c), *OfGLO1* had the highest expression in the petals (the second round of floral organs), followed by the stamens (the third round of floral organs). *OfGLO1* expression could be barely detected in the pistil, sepal, receptacle, and non-floral organs, which included the roots, leaves, and stem (Figure 3c), indicating that *OfGLO1* is a gene with floral-organ-specific expression. Overall, these observations support *OfGLO1*’s potential function as a B-class gene for floral organ determination in *O. fragrans*.

### 3.4. Phenotypic Analysis of OfGLO1 Gene-Transformed Tobacco Plants

Twenty-five tobacco plants were used for *Agrobacterium*-mediated transformation of *OfGLO1*. The expression of *OfGLO1* was detected in 16 transgenic tobacco seedlings (Figure S3). The expression level of *OfGLO1* was nearly undetectable in wild-type plants but largely increased in the tested transgenic plants (Figure S4), indicating that *OfGLO1* had been integrated into the tobacco genome and expressed successfully. Compared with the wild type, transgenic lines showed differences in flowering time, plant height, and floral organ phenotype (Table 1). First, transgenic tobacco plants flowered much earlier than the wild type, but the duration of the flowering process did not change. In the early growth stage (first 30 days) after transplanting, the transgenic tobacco lines displayed no obvious growth difference from the wild type. After 50 days of transplanting, about one-third of the transgenic tobacco plants began to develop flower buds, while the wild type was still in the vegetative growth phase. At 70 days, the flower bud development rate in transgenic tobacco reached 100%, while the wild type remained at zero. Only at 90 days after transplanting did the wild-type tobacco start developing flower buds. In addition, compared with the wild type, the plant height of transgenic tobacco was also reduced obviously, accompanied by narrower leaves, while the internode length and branch numbers increased. The average plant height of transgenic tobacco was about 10 cm shorter than that of the wild type. The length of the internode and the number of branches in the transgenic lines were around two and four times more, respectively, than that of the wild type. Furthermore, the number of leaves also decreased in the transgenic lines, while the leaf aspect ratio increased, and the leaf shape changed from an egg shape to lanceolate.

**Figure 4:**
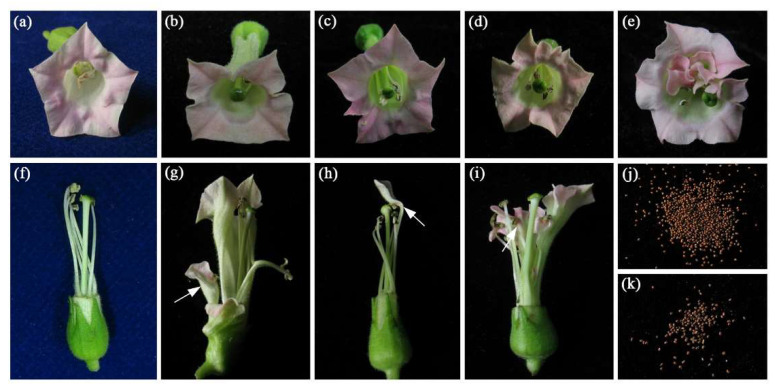
New phenotype of flowers observed in *OfGLO1*-transformed tobacco. (**a**) Normal flower of the wild type with five sepals, five petals, five stamens, and one pistil. (**b**) A flower with four petals. (**c**) A flower with six petals. (**d**) A flower with eight petals. (**e**) A stamen that became petal-like. (**f**) Lateral view of a wild-type flower, in which the petals had been removed, showing that the pistil is shorter than the stamens. (**g**) The corolla cracked, and the sepals became petal-like (indicated with a white arrow). (**h**) The white arrow shows that one stamen became petal-like in a transgenic flower. (**i**) The white arrow shows that three stamens became petal-like in a transgenic flower. (**j**) Seeds from wild-type plants. (**k**) The transgenic plants yielded less seeds.

Three new types of flower shapes were observed in the transgenic tobacco plants (Figure 4 and Table 2). Normally, the flowers of wild-type tobacco have five petals (Figure 4a). In the first new flower type, 7.5% of flowers in the transgenic plants had less than five petals (Figure 4b and Table 2); in the second flower type, more petals were observed in the transgenic plants, with six- and eight-petal flowers accounting for 15% (Figure 4c,d and Table 2); and in the third new flower type, the transgenic stamen became a petal-like structure, which accounted for 12.5% of the flowers (Figure 4e and Table 2). These observations confirmed that *OfGLO1* plays a role in the formation of the second and third rounds of floral organs, which was consistent with the qRT-PCR results that *OfGLO1* is highly expressed in the second and third rounds of floral organs (Figure 3).

In addition, the average corolla diameter in transgenic tobacco was 2 cm, which was similar to that in the wild type, while the number of floral buds was lower in transgenic tobacco than that in the wild type (Table 1). A few sepals became petals in transgenic tobacco (Figure 4g). The average pistil length in transgenic tobacco was 4.18 cm, which was also similar to that in the wild type, while the transgenic stamen length was about 0.3 cm shorter than that in the wild type, which led to the pistils in transgenic tobacco being 2–5 mm longer than the stamens (Table 1).

During the growth and development of transgenic tobacco plants, we also found that the stigma in transgenic tobacco matured earlier than the anthers and that the stamen in a few flowers changed into a petal-like structure on the filament (Figure 4h,i). Notably, at the late flowering stage, transgenic tobacco plants showed a dramatic decline in the seed setting rate. The seed setting rate in transgenic tobacco was about 11%, compared with 70% in the wild type (Table 1 and Figure 4j,k). In addition, the seed germination rate in the wild-type plants was also much lower than that in the transgenic tobacco plants (Table 1). Taken together, the above results showed that the overexpression of *OfGLO1* in transgenic tobacco significantly affected the development of flowers and seeds.

## 4. Discussion

The B-class MADS-box gene is a key regulatory factor for the determination of petals and stamens. Its original function is in the determination of plants [36]. In addition to this important function, B-class genes have also been studied as an example of gene functional differentiation (new functionalization and sub-functionalization), which included gene evolution by repetition or frameshift [9,37]. A phylogenetic analysis showed that B-class genes in angiosperms have evolved into two lineages: AP3/DEF and PI/GLO, based on their highly conserved sequence of the C terminal. The AP3/DEF gene was further divided into three branches: euAP3, paleoAP3, and TM6. The C terminal region of the *euAP3* gene has a PI derivative motif (FxFRLOPSQPNLH) and an euAP3 motif (SDLTTFALLE). The C terminal region of the *TM6*-type gene and the *paleoAP3*-type gene have a PI derivative motif and a paleoAP3 motif (YGxHDLRLA). In comparison, the C terminal region of the PI-type gene only has a PI motif (MPFxFRVQPxPNLQE) [38]. The function of the B-class gene in floral development has been studied in various plants, but its expression in *O. fragrans* has not been reported. Based on the partial CDS sequence of Osmanthus GLO1, the full-length cDNA sequence was cloned using the RACE technique in this study (Figure 1 and Figure S1). A sequence analysis indicated that the C terminal of *OfGLO1* contained PI motifs (Figure 2), thus supporting *OfGLO1* as the PI-type B-class MADS-box gene.

The classical ABC model suggests that the B-class genes are generally expressed only in the second and third rounds of floral organs. Our results in this study (Figure 3) are exclusively consistent with this prediction. However, the temporal and spatial expression patterns of PI/GLO homologous genes are different across plants. In *Arabidopsis*, the flower developed to the third stage, PI was highly expressed in the second and third rounds of floral organs, and a little expression was found in the carpel primordia (fourth round). However, after entering the fifth stage, PI is only expressed in the petals and stamens [16]. The *GLO* of snapdragon can be detected from the appearance of sepal primordium. It was expressed in the petals and stamens in high abundance and had a small amount of expression in the development of the carpels [14]. The GLO homologous genes of monocotyledons such as rice (*Oryza sativa*) [17], *Zea mays* [18], and *Asparagus officinalis* [20], which are significantly different from petals to calyx, are also consistent with the ABC model prediction. However, this model does not apply to monocotyledons with petal-like sepals. The expression pattern of *LLGLO1* in *Lilium longiflorum* [22] and *CfGLO* in *Cymbidium faberi* [23] showed that the B-class genes were expressed not only in petals and stamens but also in sepals. Considering the fact that the GLO gene is expressed in the fourth round of the floral organ in many angiosperms while the *GLO* mutation does not affect the identities of the carpels, we can propose that the function of the ancestral gene may be lost while still retaining its expression in carpel.

In addition, B-class genes also have a variety of other functions. For example, the *CjGLO1* and *CjGLO2* in *Camelia japonica* 'Shibaxueshi' were involved in the formation of the pleiopetalous flower [39], and *CiGLO* [26] in *Canna indica* helped convert the stamen into a petal-like structure. In this study, the expression levels of *OfGLO1* were different in the different flower development stages (Figure 3). *OfGLO1* has the highest expression at the full-flowering stage, and during this stage, *OfGLO1* was highly expressed only in the second and third rounds, while no expression was detected in the fourth round and in other non-floral organs (Figure 3c). This result confirmed those of the classical ABC model, indicating that *OfGLO1* was involved in the developmental regulation of petals and stamens in *O. fragrans*.

*OfGLO1*-transformed tobacco showed greatly reduced plant heights, longer internode, earlier flowering, an increased number of petals, shorter stamens, stamens that were partially changed into petal-like structures, and stigmas that were longer than the stamens by 2–5 mm (Figure 4 and Table 1 and Table 2). These results were based on the phenotyping of sixteen independent T_0_ *OfGLO1* transgenic lines and were supported by statistical analyses. The overexpression of *OfGLO1* in T_0_ lines was verified by semi RT-PCR and qRT-PCR, suggesting that the observed phenotypes were most likely caused by *OfGLO1* over-expression. Our study reveals that *OfGLO1* was involved in the regulation of the flowering time and in floral organ development. The transgenic plants flowered much earlier in this study, possibly because of the overexpression of *OfGLO1* activating some flowering pathway integrators (such as *LFY*, *SOC1*, etc.). According to the boundary sliding model, the sepals of transgenic tobacco partially changed into petal-like structures, possibly due to the expression domain of *OfGLO1* being extended to the first round of floral organs. In transgenic tobacco, the stamens also changed into petal-like structures, which may be because the overexpression of the *OfGLO1* gene in the third round of floral organs weakened the function of C-class genes. Despite this, we believe that the current phenotyping results are generally reliable; further phenotype verification may be performed using T1 and T2 lines, which are considered to have a more stable gene expression. In addition, due to the recent publication of the reference genome of *O. fragrans* [29], flower development related genes in *O. fragrans* could be further studied in a systematic manner to allow us more insights into flower development in this important tree species.

## 5. Conclusions

In summary, we cloned the full-length cDNA of *OfGLO1* and investigated its potential role in floral organ development. We showed that *OfGLO1* plays a role in the regulation of flower transition and in petal and stamen formation.

## Figures and Tables

**Table 1:** Comparison of the characteristics between the *OfGLO1*-transformed and wild-type plants.

Characteristics	Wild Type	*OfGLO1*-Transformed
Differentiation rate of floral bud at 70 days	0.00	100.00 *
Plant height (cm)	83.90	73.63 *
Leaf number	30.50	25.75 *
Length–width ratio of leaf	2.06	2.31 *
Internode length (cm)	2.45	4.48 *
Branching number	2.25	8.38 *
Floral bud number	25.00	21.63 *
Stamen length (cm)	4.20	3.86 *
Pistil length (cm)	4.16	4.18
Corolla diameter (cm)	2.00	1.98
Seed setting rate (%)	70.00	11.25 *
Seed germination rate (%)	96.25	28.75 *

The data were calculated at the full flowering stage, and the average value is presented. Eight wild-type plants and sixteen T_0_ *OfGLO1*-overexpressed tobacco plants were used for characteristic measurement. The T_1_ generation seeds (*n* = 80) were used for the seed germination rate test. Asterisks (*) indicate a significant difference between the *OfGLO1*-transformed and wild-type plants at *p* < 0.05, as determined by Student's *t* tests.

**Table 2:** Three new flower types in *OfGLO1*-transformed tobacco.

New Phenotypes of Flower	No. of Total Flowers	Flower No. with New Phenotype	Percentage (%)
No. of petals was less than 5	80	6	7.5
No. of petals was more than 5	80	12	15
Stamen became petal-like	80	10	12.5

The statistics were calculated 70 days after the in vitro seedlings were transplanted.

## Data Availability

The data presented in this study are available in the manuscript and in the Supplementary Materials.

## Supplementary Materials

The following are available online at https://www.mdpi.com/article/10.3390/genes12111748/s1, Figure S1: The flowers of *O. fragrans* in the early flowering stage. Figure S2: Agarose gel electrophoresis of DNA fragments. Figure S3: RT-PCR detection of tobaccos transformed with the *OfGLO1* gene. Figure S4: The expression level of *OfGLO1* in transgenic tobacco leaves was detected using quantitative real-time RT-PCR. Figure S5: The gene structure analysis of *OfGLO1*. Gray line, intron; black box, exon. Table S1: Primers used for cloning, vector construction, and expression analysis of *OfGLO1* in *O. fragrans*. Table S2: Conserved elements in the promoter of *OfGLO1*, *BpMADS2* and *AtGLO1*.
