# Supplementary material for: Cloning, Expression, and Tobacco Overexpression Analyses of a PISTILLATA/GLOBOSA-like (OfGLO1) Gene from Osmanthus fragrans
Source: Genes (Basel). 2021 Oct 30;12(11):1748. doi: 10.3390/genes12111748 (PMC8623234; doi:10.3390/genes12111748)
Supplement: Supplementary file 1 [file genes-12-01748-s001.zip › genes-1406333-supplementary.pdf]

Supplementary Materials

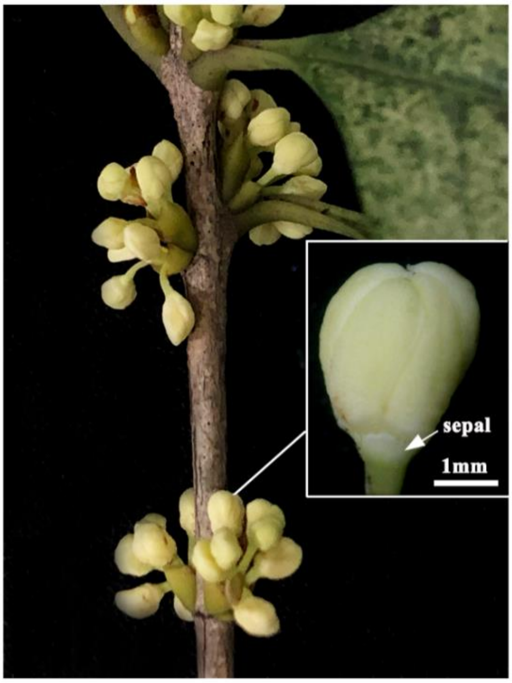

Figure S1. The flowers of *O. fragrans* in the early flowering stage. White arrow showed the sepal structure. Bar, 1mm.

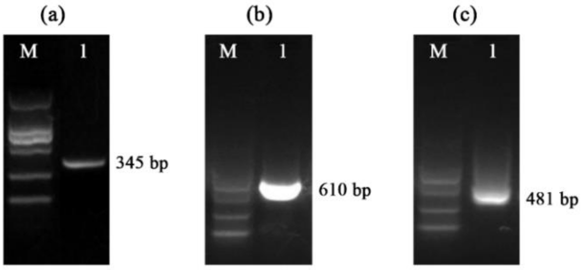

Figure S2. Agarose gel electrophoresis of DNA fragments. The cloning results of the middle fragment (a), 3' RACE (b), 5' RACE (c) of *OfGLO1* gene. M: DM 2000 DNA marker; 1: Target fragment.

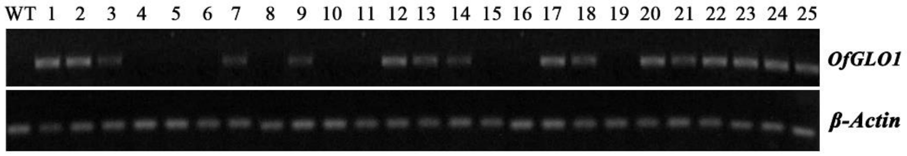

Figure S3. RT-PCR detection of tobaccos transformed with the *OfGLO1* gene. WT: wild-type.

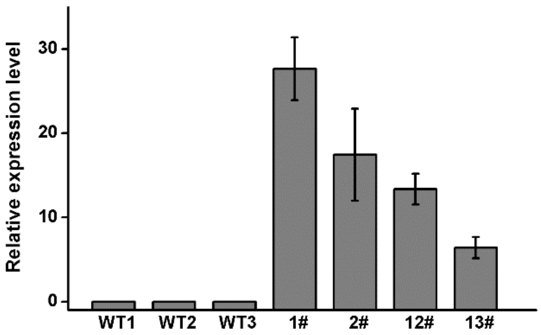

Figure S4. The expression level of *OfGLO1* in transgenic tobacco leaves was detected using quantitative real-time RT-PCR. Cytokine gene (*β-Actin*) was used as the internal reference gene. WT: wild-type.

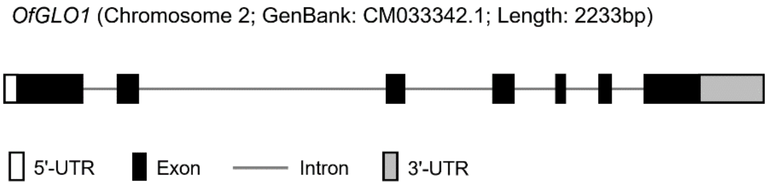

Figure S5. The gene structure analysis of *OfGLO1*. Gray line, intron; black box, exon.

Table S1. Primers used for cloning, vector construction and expression analysis of *OfGLO1* in *O. fragrans*

**Primer purpose**	**Primer name**	**Primer sequence (5' - 3')**
The middle fragment cloning	GLO1-F	GCATGAGCATTTGGACAA
	GLO1-R	GGCATTTGGTATTCGTAGTC
Detecting positive colony	M13-F	GTAAAACGACGGCCAGT
	M13-R	CAGGAAACAGCTATGAC
3' RACE	GLO1-3'-1	GGAAGAAGCTCTGGGATGCTAA
	GLO1-3'-2	GTCTGAGTGGAGAGGACATAACAA
	Outer Primer	GCGAGCACAGAATTAATACGACT
	Inner Primer	CGCGGATCCGAATTAATACGACTCACTATAGG
5' RACE	GLO1-5'-1	CCTCATCATCGGCACACACT
	GLO1-5'-2	GAGTTCAGTCTGCATCCTGTCATT
	Outer Primer	GCTGATGGCGATGAATGAACACTG
	Inner Primer	CGCGGATCCGAACACTGCGTTTGCTGGCTTTGATG
cDNA cloning and expression	GLO1-Kpn Ⅰ	GGGGTACCATGGGGAGAGGGAAGAT
vetor construction	GLO1-Xba Ⅰ	GCTCTAGACTAGAAACGATCCTGTAAATTAG
Expression level analysis	qGLO1-F	CAGTACCATAAACTCTCTGGGAAG
	qGLO1-R	GTTGTTATGTCCTCTCCACTCAG
	qActin-F	ATTATTTCCTTGCTCATACGGTCAG
	qActin-R	ATTAGTCCTCTTCCAGCCTTCTTTG
	β-Actin-F	GATCTTGCTGGTCGTGATCT
	β-Actin-R	ACTTCCGGACATCTGAACCT

Note: ORF, open reading frame.

Table S2. Conserved elements in the promoter of *OfGLO1*, *BpMADS2* and *AtGLO1*.

Species	Homolog genes of *PI*/*GLO*	Putative motifs	Nucleotide sequence	Location (relative to ATG)
Osmanthus fragrans	*OfGLO1* (KY575409)	putative CArG-box	CCTATTTCTGG	-2775 to -2765
			CCACTAAATGG	-2501 to -2491
			CCTCTTTTGG	-1427 to -1418
			CCTACAATGG	-153 to -144
		PI late expression element	TCAAGAAA	-2834 to -2827; -2165 to -2158; -650 to -643
Betula pendula Roth	*BpMADS2* (AJ968321)	putative CArG-box	CCTTGTTTAGG	-2993 to -2283
			CCATAAAAGG	-2207 to -2198
			CCATGTTTGG	-2158 to -2149
			CCTTAGTTTGG	-1722 to -1712
Arabidopsis	*AtGLO1* (AT5G20240)	putative CArG-box	CCTCATTAGG	-1856 to -1847
		PI late expression element	TCAAGAAA	-1197 to -1190; -210 to -203

Note: *PI*, *PISTILLATA*; *GLO*, *GLOBOSA*. ATG, translation initiation codon.
